# SALL4 ‐ KHDRBS3 network enhances stemness by modulating CD44 splicing in basal‐like breast cancer

**DOI:** 10.1002/cam4.1296

**Published:** 2018-01-22

**Authors:** Yoshiaki Matsumoto, Junji Itou, Fumiaki Sato, Masakazu Toi

**Affiliations:** ^1^ Department of Breast Surgery Graduate School of Medicine Kyoto University Kyoto Japan

**Keywords:** Breast neoplasms, protein isoforms, RNA splicing factors, stem cell factor, transcription factors

## Abstract

Understanding the mechanism by which cancer cells enhance stemness facilitates cancer therapies. Here, we revealed that a stem cell transcription factor, SALL4, functions to enhance stemness in basal‐like breast cancer cells. We used shRNA‐mediated knockdown and gene overexpression systems to analyze gene functions. To evaluate stemness, we performed a sphere formation assay. In SALL4 knockdown cells, the sphere formation ability was reduced, indicating that SALL4 enhances stemness. CD44 is a membrane protein and is known as a stemness factor in cancer. CD44 splicing variants are involved in cancer stemness. We discovered that SALL4 modulates CD44 alternative splicing through the upregulation of KHDRBS3, a splicing factor for CD44. We cloned the KHDRBS3‐regulated CD44 splicing isoform (CD44v), which lacks exons 8 and 9. CD44v overexpression prevented a reduction in the sphere formation ability by KHDRBS3 knockdown, indicating that CD44v is positively involved in cancer stemness. In addition, CD44v enhanced anoikis resistance under the control of the SALL4 ‐ KHDRBS3 network. Basal‐like breast cancer is an aggressive subtype among breast cancers, and there is no effective therapy so far. Our findings provide molecular targets for basal‐like breast cancer therapy. In the future, this study may contribute to the establishment of drugs targeting cancer stemness.

## Introduction

A cancer cell having stemness can generate tumor tissue, which facilitates the recurrence and formation of metastatic focus. Therefore, understanding how cancer cells enhance their stemness may contribute to cancer therapies.

To evaluate cancer stemness, a sphere formation assay with an ultralow attachment dish is widely used. In a floating condition with an ultralow attachment dish, a solid cancer cell cannot survive for a long time; this phenomenon is called anoikis. On the other hand, a cancer cell having stemness has anoikis resistance and tumor formation ability, which results in survival, proliferation, and formation of a sphere in a floating condition.

Among breast cancer subtypes, basal‐like breast cancer has high stemness 1. A membrane‐bound hyaluronan receptor CD44 is known as a factor for stemness 2. In humans, the CD44 gene has various isoforms that are generated by alternative splicing of 9 variant exons 3. The CD44 standard form has an extracellular ligand‐binding region, a transmembrane region, and an intracellular region; however, this form does not contain variant exons. CD44 variant isoforms have insertions of variant exons between the extracellular and the transmembrane regions. The expressions of CD44 variant isoforms are used for cancer stem cell markers, as well as upregulation of CD44 expression 2, 4, 5. However, not all CD44 isoforms involved in stemness have been identified.

Previous studies have identified some splicing factors for CD44 isoform expression, such as ESRP1, hnRNPM, Tra2‐*β*1, and KHDRBS3 5, 6, 7, 8, 9. ESRP1 regulates the expression of variant exons 5, 6. In contrast, hnRNPM inhibits the inclusion of the variant exons in CD44 mRNA 7. Tra2‐*β*1 increases the expression of exons 8 and 9 8. KHDRBS3 (KH RNA Binding Domain Containing, Signal Transduction Associated 3) has been identified as a regulator for CD44 exon 9 9. However, the regulation of CD44 alternative splicing has not been fully understood in basal‐like breast cancer.

Developmental transcription factor SALL4 has been identified as a factor for cancer cell proliferation in various types of cancers mainly though the upregulation of a transcriptional repressor of *INK4* genes, BMI‐1 10, 11. Recent papers showed a correlation between SALL4 expression and poor survival rate in patients having breast cancer and other kinds of cancers 12, 13. In breast cancer, SALL4 expression was observed in the tissues from breast cancer patients 14, and its mRNA level was higher in basal‐like subtype than in other subtypes in the cancer genome atlas database 15, 16. SALL4 is also expressed in basal‐like breast cancer cell lines. Therefore, to analyze SALL4 function, shRNA‐mediated knockdown experiments were conducted in previous studies 11, 16. In basal‐like breast cancer, in addition to cell proliferation, SALL4 promotes cell migration for metastasis by upregulating the expression of integrin genes 16. In addition, previous studies have reported that SALL4 is positively involved in tumor formation in liver cancer 17, 18. These suggest that SALL4 promotes cancer malignancy. However, how SALL4 augments stemness remains elusive.

Here, we found that SALL4 upregulates a splicing factor, KHDRBS3. KHDRBS3 changes CD44 isoform expression. We identified a novel CD44 isoform, which is regulated by KHDRBS3 and enhances stemness. Moreover, the SALL4 ‐ KHDRBS3 ‐ CD44 isoform network augments anoikis resistance. Our findings provide a novel mechanism to enhance stemness in breast cancer. This study may contribute to the development of cancer therapies targeting stemness.

## Materials and Methods

### Cell culture

Basal‐like breast cancer cell lines, SUM159 and Hs578T, were obtained from Asterand (Detroit, MI, USA) and the American Type Culture Collection (Manassas, VA, USA), respectively. SUM159 cells were maintained with Ham's F‐12 nutrient mixture containing 5% FBS, 5 *μ*g/mL insulin, 1 *μ*g/mL hydrocortisone, and 10 mmol/L HEPES. Hs578T cells were cultured with DMEM containing 10% FBS. For the cell growth assay, cells were plated with the density of 50,000 cells/well, and the cell number was manually counted after culturing.

### Gene knockdown and overexpression systems

Gene knockdown was performed using the pLKO vector (Addgene, 8453, Cambridge, MA, USA). The double‐strand DNA oligo with shRNA sequence was inserted. The target sequences of shRNAs are listed in Supporting table S1. Knockdown cells were used 6 days after infection. For gene overexpression, pLenti 6.3 vector (Life Technologies, V533‐06, Carlsbad, CA, USA) was used. The overexpression gene was cloned into the pENTR‐FLAG vector 19, and subcloned into the pLenti 6.3 vector.

### Sphere formation assay

For the sphere formation assay, 1000 cells were cultured in a well of an ultralow attachment 24‐well plate (Corning, 3473, Kennebunk, ME, USA) with 500 *μ*L of DMEM/F‐12 medium containing 10 ng/mL EGF, 10 ng/mL basic FGF, 1% B‐27, 5 *μ*g/mL insulin and 0.03% LA717 (Wako, 381‐09041, Osaka, Japan). After 16 days, a whole image of each well was taken and the number of spheres larger than 50 *μ*m was manually counted using ImageJ software.

### RNA sampling and reverse transcription PCR

Total RNA sample was extracted with TRIzol reagent (Thermo Fisher Scientific, 15596026, Waltham, MA, USA). A complementary DNA sample was synthesized with SuperScript III reverse transcriptase (Thermo Fisher Scientific, 18080044) and dT18 primer. Real‐time PCR was performed with the primers listed in Supporting table S2. Relative mRNA level was calculated by ddCt method. The sequences of primers for the CD44 variant analysis are shown in Supporting table S3.

### Immunoblotting

The primary antibodies for immunoblotting are anti‐FLAG antibody (Wako, 018‐22386, 1/1000 dilution), anti‐CD44 antibody (Thermo Fisher Scientific, MS‐668‐P0, 1/1000 dilution), and anti‐*β*‐actin antibody (Abcam, ab6276, Cambridge, UK, 1/5000 dilution). Rather than using a secondary antibody to detect FLAG and CD44 signals, the Easy‐Western‐II detection system (Beacle, BCL‐EZS21, Kyoto, Japan) was employed. The secondary antibody to detect *β*‐actin signal was goat anti‐mouse IgG antibody conjugated to a peroxidase (Pierce biotechnology, 31340, Rockford, IL, USA, 1/50000 dilution).

### Anoikis resistance assay

Anoikis resistance was analyzed using a kit, CytoSelect 96‐well Anoikis Assay (Cell Biolabs, Inc., CBA‐081, San Diego, CA, USA). We used 50,000 cells per well, and cultured the cells for 24 h. Calcein AM and EthD‐1 staining was performed according to the manufacturer's instructions. Quantification of anoikis resistance with MTT assay was performed according to the manufacturer's instructions.

### Statistical analysis

We used Student's *t*‐test for statistical analyses. *P *<* *0.05 was considered statistically significant. Error bars show standard deviations.

## Results

### SALL4 enhances stemness in basal‐like breast cancer cells

**Figure 1:**
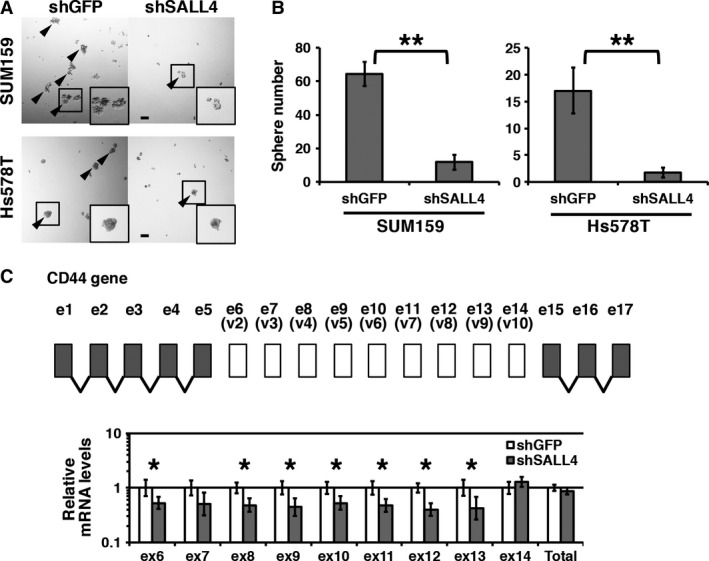
SALL4 enhances stemness and modulates the expression of CD44 variant exons. (A) Spheres of the control and SALL4 knockdown cells are shown. SUM159 and Hs578T cells were used. Arrowheads indicate spheres larger than 50 *μ*m. (B) Numbers of spheres are graphed. (C) Quantification of the expression of CD44 variant exons and total CD44 is shown. Gene structure of CD44 is depicted. The graph shows relative mRNA levels to the shGFP control. Scale bar: 100 *μ*m. *P <* *0.05, **P <* *0.01.

To determine whether SALL4 is involved in stemness, we used the shRNA‐mediated knockdown system 16 and performed a sphere formation assay. In a sphere formation assay, if cells aggregate each other, large clusters are observed even if cells do not have sphere formation ability. That causes inaccurate results. To avoid cell aggregation during culturing, we added the LA717 resin, which blocks aggregation through prevention of cell movement in a floating condition. When we seeded cells uniformly, each sphere was generated from a single cell without aggregation. In this condition, cell clusters did not grow to a large size, and we determined cell clusters larger than 50 *μ*m in diameter as spheres not generated by aggregation. We used a basal‐like breast cancer cell lines SUM159 and Hs578T, and observed reduced sphere formation ability in SALL4 knockdown cells (Fig. 1A). To quantify the difference in sphere formation ability, we counted the number of spheres larger than 50 *μ*m. In the results, the sphere formation ability of SALL4 knockdown cells was significantly reduced, compared to that of the shGFP control (Fig. 1B), indicating that SALL4 enhances stemness.

In breast cancer, a change in CD44 alternative splicing is involved in stemness 5. The human CD44 gene has 9 variant exons that are from exon 6 to 14 (also known as variant exon 2 to 10) 20. We therefore quantified the expression levels of the CD44 variant exons and total CD44 (Fig. 1C). We observed a reduction in the mRNA levels of some variant exons in SALL4 knockdown cells, whereas the total CD44 mRNA level was not significantly altered by SALL4 knockdown. These results suggest that SALL4 modulates CD44 splicing for stemness.

### SALL4 regulates KHDRBS3 gene for stemness

**Figure 2:**
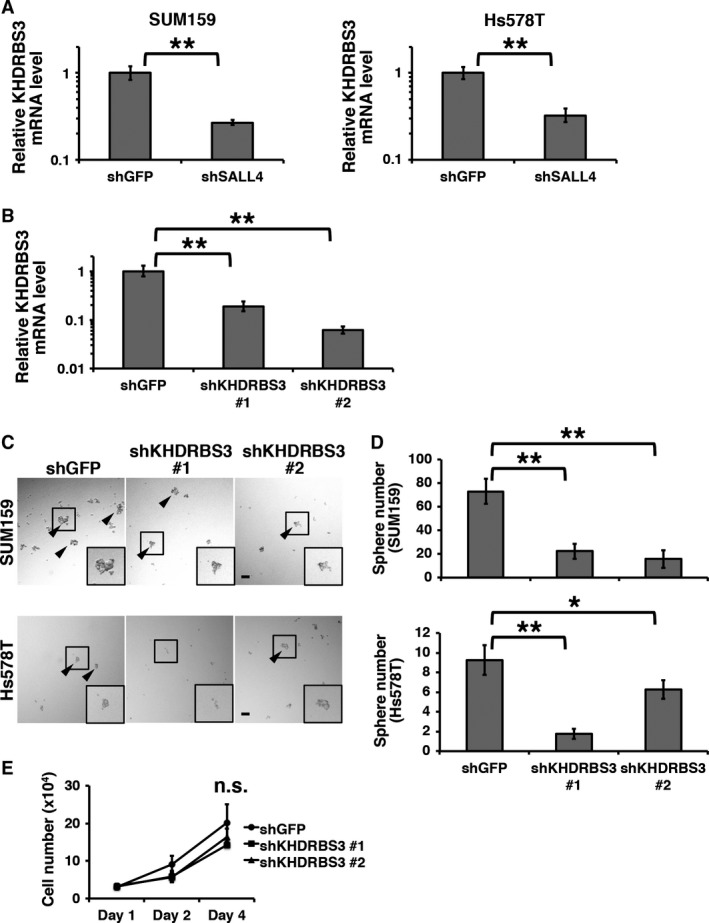
KHDRBS3 enhances stemness under the control of SALL4. (A) Relative mRNA levels of KHDRBS3 to the shGFP control are shown. SUM159 and Hs578T cells were used. (B) The efficiency of shRNA‐mediated KHDRBS3 knockdown systems is shown. (C) Spheres of the control and KHDRBS3 knockdown cells are shown. Arrowheads indicate spheres larger than 50 *μ*m. (D) Numbers of spheres are graphed. (E) Cell numbers of the control and KHDRBS3 knockdown cells are shown. Scale bar: 100 *μ*m. *P <* *0.05, **P <* *0.01.

Given that SALL4 is a transcription factor, we hypothesized that SALL4 regulates the expression of a splicing factor for CD44 mRNA. In the previously published list of SALL4‐regulated genes 16, we found the KHDRBS3 gene, the product of which is known as a splicing factor for CD44 9. We analyzed the mRNA level of the KHDRBS3 gene in SALL4 knockdown cells and observed a reduction in KHDRBS3 expression compared to that of the shGFP control (Fig. 2A), indicating that SALL4 upregulates KHDRBS3 expression. We performed a co‐occurrence analysis between SALL4 and KHDRBS3 genes in the RNA‐seq data from breast cancer patients published by the cancer genome atlas 21 in the cBioportal platform 22. The result showed that SALL4 and KHDRBS3 expressions co‐occurred significantly (*P *=* *0.011, Fisher's exact test), which supports our findings.

To determine whether KHDRBS3 is involved in stemness, we constructed a KHDRBS3 knockdown system (Fig. 2B) and performed sphere formation assays. In the results, the sphere numbers of KHDRBS3 knockdown cells were significantly smaller than that of the controls (Fig. 2C). We suspected that if KHDRBS3 knockdown inhibits cell proliferation, KHDRBS3 knockdown cells would show a smaller number of spheres than the control cells even if the stemness is not changed. Therefore, we analyzed cell growth and observed no significant difference between the shGFP control and KHDRBS3 knockdown cells. These results indicate that KHDRBS3 enhances stemness in basal‐like breast cancer cells.

### The SALL4 ‐ KHDRBS3 network enhances stemness

**Figure 3:**
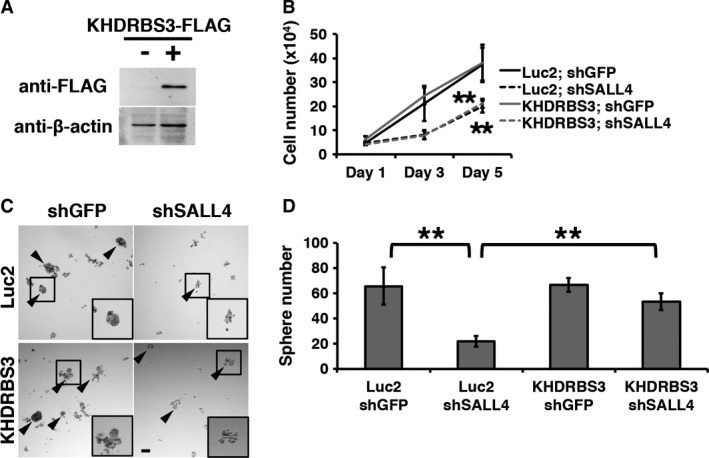
The SALL4 ‐ KHDRBS3 network enhances stemness. (A) KHDRBS3‐overexpressing cells were established. Immunoblotting images are shown. (B) The results of cell growth assay are shown. Student's *t*‐test was performed against the values of Luc2; shGFP cells. (C) Spheres of the control and SALL4 knockdown cells are shown. Arrowheads indicate spheres larger than 50 *μ*m. (D) Numbers of spheres are graphed. Scale bar: 100 *μ*m. **P <* *0.01.

To analyze the function of the SALL4 ‐ KHDRBS3 network on stemness, we established KHDRBS3 overexpression cells in SUM159 cells (Fig. 3A). We used luciferase2 (Luc2) overexpression as the control. SALL4 knockdown reduced cell growth as previously reported 11, 18, and KHDRBS3 overexpression was not able to restore it (Fig. 3B), indicating that KHDRBS3 is not involved in cell growth. We performed a sphere formation assay with KHDRBS3‐overexpressing cells. KHDRBS3 overexpression did not increase the sphere formation ability of shGFP cells (Fig. 3C,D). Reduced sphere formation ability was observed in SALL4 knockdown cells, and KHDRBS3 overexpression restored it (Fig. 3C,D), indicating that the SALL4 ‐ KHDRBS3 network enhances stemness.

### The SALL4 ‐ KHDRBS3 network alters CD44 splicing for stemness

**Figure 4:**
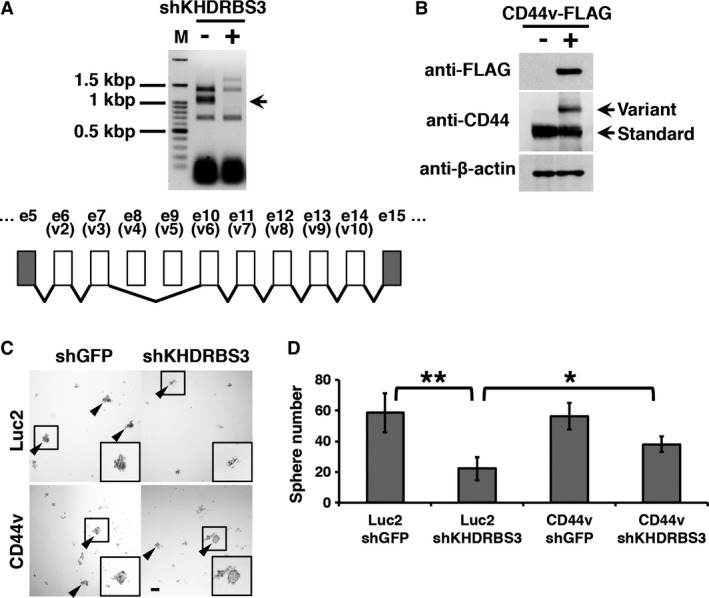
KHDRBS3‐modulated CD44 isoform enhances stemness. (A) An agarose gel electrophoresis image of CD44 variant expression is shown. Arrow indicates the isoform on which we focused in this study (CD44v). The structure of CD44v is depicted. (B) CD44v‐overexpressing cells were established. Immunoblotting images are shown. (C) Spheres of the control and KHDRBS3 knockdown cells are shown. ShKHDRBS3 #1 was used. Arrowheads indicate spheres larger than 50 *μ*m. (D) Numbers of spheres are graphed. Scale bar: 100 *μ*m. *P <* *0.05, **P <* *0.01.

CD44 has several splicing isoforms with various combinations of variant exons 2. To identify the isoform regulated by KHDRBS3 in basal‐like breast cancer, we performed reverse transcription PCR with forward and reverse primers for CD44 designed at exon 5 and 15, respectively. The expression pattern of CD44 isoforms was changed by KHDRBS3 knockdown (Fig. 4A). Among these isoforms, we focused on an isoform of which the expression level was reduced to undetectable levels by KHDRBS3 knockdown (Fig. 4A, arrow). This isoform does not contain exon 8 and 9 (hereinafter, referred to the isoform as CD44v) (Fig. 4A).

To determine whether CD44v acts as a stemness factor under the control of the SALL4 ‐ KHDRBS3 network, we introduced CD44v overexpression into SUM159 cells (Fig. 4B) and performed a sphere formation assay in combination with KHDRBS3 knockdown experiments. In the Luc2 control cells, KHDRBS3 knockdown reduced sphere formation ability (Fig. 4C,D). This reduction was not observed in CD44v‐overexpressing cells (Fig. 4C,D). These results suggest that the SALL4 ‐ KHDRBS3 network enhances stemness through the regulation of CD44 splicing.

### The SALL4 ‐ KHDRBS3 ‐ CD44v network enhances anoikis resistance

**Figure 5:**
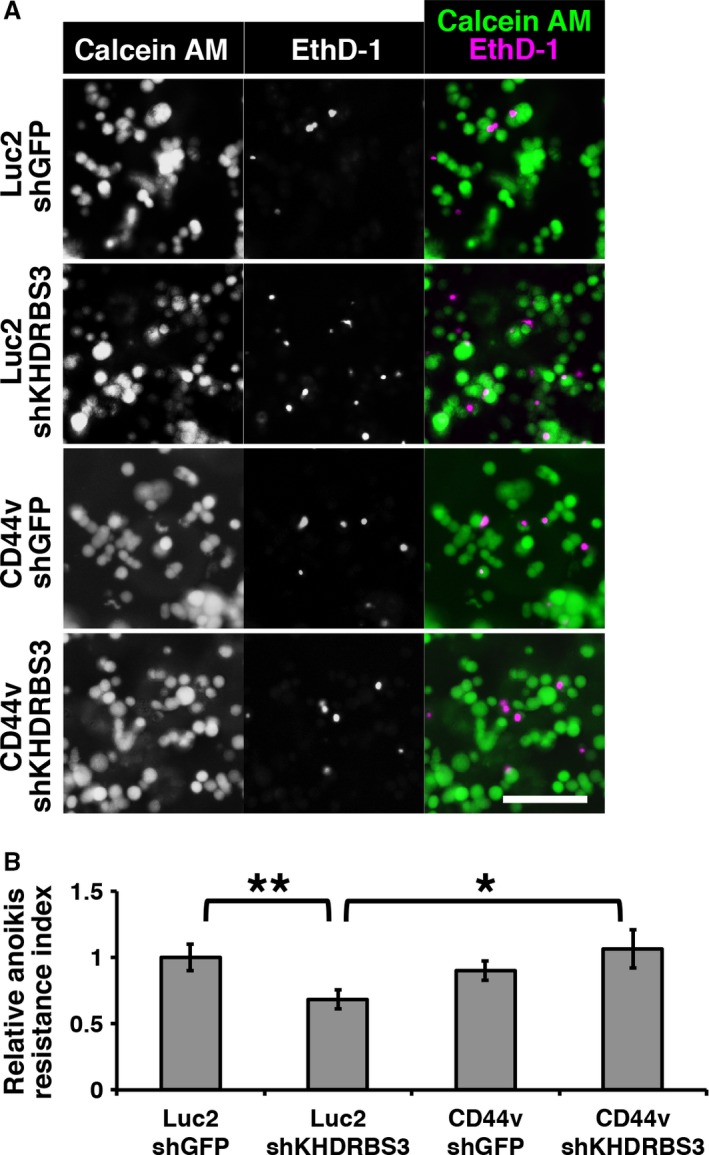
KHDRBS3‐modulated CD44 isoform enhances anoikis resistance. (A) Images of fluorescent staining for living cells (stained with Calcein AM) and dead cells (stained with EthD‐1) are shown. ShKHDRBS3 #1 was used. (B) Quantification of living cells after 24 h culturing is shown. Living cells were quantified with MTT assay. Scale bar: 100 *μ*m. *P <* *0.05, **P <* *0.01.

Anoikis resistance is one of the hallmarks of stemness in cancer and is required for sphere formation. To determine whether the SALL4 ‐ KHDRBS3 ‐ CD44v network enhances anoikis resistance, we performed an anoikis assay in the control cells and CD44v‐overexpression cells with or without KHDRBS3 knockdown. In the results, EthD‐1‐stained dead cell signals were increased by KHDRBS3 knockdown in Luc2 control cells (Fig. 5A). However, such signals were not increased by KHDRBS3 knockdown in CD44v‐overexpressing cells (Fig. 5A). To evaluate anoikis resistance, we quantified living cell values with an MTT assay. Reduced anoikis resistance by KHDRBS3 knockdown was not observed in CD44v‐overexpressing cells (Fig. 5B), suggesting that the SALL4 ‐ KHDRBS3 ‐ CD44v network provides stemness through enhancement of anoikis resistance.

In our experiments (Fig. 5), CD44v overexpression did not increase anoikis resistance in the shGFP control cells. This suggests that although CD44v is required for anoikis resistance, it is not a factor to enhance anoikis resistance. Another suggestion is that this cell line already has the maximum level of anoikis resistance, and CD44v overexpression could not increase it.

## Discussion

Cancer stemness is one of the factors for recurrence and formation of a metastatic focus. In this study, we showed that SALL4 enhances stemness and modulates the expression of CD44 variant exons through upregulation of KHDRBS3 expression. We identified the CD44 isoform under the control of the SALL4 ‐ KHDRBS3 network. Overexpression of the isoform (called CD44v in this study) restored the reduced sphere formation ability by KHDRBS3 knockdown, indicating that the SALL4 ‐ KHDRBS3 network‐regulated CD44v enhances stemness. In addition, we discovered that CD44v expression augments anoikis resistance.

In gastric cancer, SALL4 upregulates CD44 expression 23. However, we observed no significant change in the total CD44 expression level by SALL4 knockdown, indicating that SALL4 does not regulate CD44 transcription in basal‐like breast cancer cells. This difference suggests that the regulatory mechanism of CD44 expression differs between gastric cancer cells and basal‐like breast cancer cells.

CD44 has splicing isoforms 2. CD44 variants contain variant exon(s) in their mRNA, while CD44 standard does not have variant exons. Among CD44 variants, the isoform having exon 10 is known as a stemness factor in cancer 24. In a non‐metastatic rat pancreatic carcinoma cell line, overexpression of an exon 10‐containing isoform of CD44 provided a metastatic potential 25. Anti‐CD44 exon 10 antibody blocked metastasis 26. Patients having colorectal cancer with high expression of CD44 exon 6 had a poor prognosis 27. This study identified a novel splicing isoform, CD44v, which does not have exon 8 and 9. Although this isoform had not been identified as a stemness factor, it contains exons 6 and 10, which is consistent with previous studies 24, 27.

In cancer, it has been reported that Serpin B5 interacts with KHDRBS3 and FBXO32 in gastric cancer and acts as an oncogenic factor 28. However, the function of KHDRBS3 on cancer stemness remained unknown. We showed that KHDRBS3 enhances stemness in basal‐like breast cancer. This study identified KHDRBS3 as a regulator for CD44v under the regulation of SALL4. This finding differs from the previous study, which reported that KHDRBS3 functions to express exon 9 9. This seems to be due to the difference in cell lines and/or conditions. Alternative splicing is regulated by a combination of splicing factors 7, 29. Given that CD44 has 9 variant exons and the regulation of the expression of these exons is complex, SALL4‐regulated KHDRBS3 expression seems to be one of the factors for CD44 variant expression in basal‐like breast cancer.

CD44 is used as a marker for stemness in breast cancer 2, 30. The expression of CD44 variant exons increases metastasis 25. However, the mechanism how CD44 contributes to stemness and metastasis has not been fully studied. Anoikis resistance is involved in both stemness and metastasis. This study focused on stemness in basal‐like breast cancer, and discovered that CD44v increases anoikis resistance. Although various factors contribute to cancer stemness and metastasis, and further study is required to understand the function of CD44, we revealed one of the regulatory mechanisms of anoikis resistance.

SALL4 is a transcription factor, and SALL4 knockdown changes various cellular events. Although some SALL4‐regulated genes involving in breast cancer malignancy have been identified, how SALL4 promotes stemness remained elusive. This study revealed that SALL4 regulates CD44v expression via KHDRBS3 for stemness, which might contribute to understanding of the function of SALL4 on cancer stemness.

In our observation, change in the pattern of the variant exon expression differed between SALL4 and KHDRBS3 knockdowns. These suggest that SALL4 knockdown influences the expression and/or function of other splicing factors. As for the stemness of basal‐like breast cancer, our KHDRBS3 overexpression experiments showed prevention of the reduction in sphere formation ability by SALL4 knockdown. This indicates that although other splicing factors might function to regulate CD44 alternative splicing under the control of SALL4, the SALL4 ‐ KHDRBS3 network contributes to stemness in basal‐like breast cancer.

Currently the major therapeutic approach for basal‐like breast cancer is chemotherapy, and there is no targeted therapy approved for this subtype of breast cancer. We discovered a novel molecular network for stemness, which could be a therapeutic target. Our findings may contribute to the development of stemness‐targeted therapy.

## Conflict of Interest

YM and FS have no conflict of interest. JI is an employee of Kyoto University's Sponsored Research Program funded by Taiho Pharmaceutical Co., Ltd. MT received research funding from Taiho Pharmaceutical Co., Ltd. The funding source had no role in the study design, experiment, analysis, interpretation or writing the manuscript.

## Supporting information


**Table S1.** Target sequences of shRNAs.
**Table S2.** Sequences for oligos for quantitative reverse transcription PCR.
**Table S3.** Sequences for analysis of CD44 isoform expression.
