# Supplementary material for: SALL4 ‐ KHDRBS3 network enhances stemness by modulating CD44 splicing in basal‐like breast cancer
Source: Cancer Med. 2018 Jan 22;7(2):454–62. doi: 10.1002/cam4.1296 (PMC5806117; doi:10.1002/cam4.1296)
Supplement: Supplementary file 1 — Table S1. Target sequences of shRNAs. Table S2. Sequences for oligos for quantitative reverse transcription PCR. Table S3. Sequences for analysis of CD44 isoform expression. [file CAM4-7-454-s001.docx]

**Supporting table S1.** Target sequences of shRNAs

| shRNA | Target sequence (5’ 🡪 3’) |
| --- | --- |
| shGFP (control) | GCACGACTTCTTCAAGTCCGC |
| shSALL4 | GTGAGGATGAAGCCACAGTAA |
| shKHDRBS3 #1 | GTGGCAATTCTCTGAAGCGTT |
| shKHDRBS3 #2 | GCTGGGACAGAAAGTGTTAAT |

**Supporting table S2.** Sequences for oligos for quantitative reverse transcription PCR

| Target | Direction | Sequence (5’ 🡪 3’) |
| --- | --- | --- |
| CD44 exon6 | Forward | CTAGTGCTACAGCAACTGAGAC |
|  | Reverse | CCATTTGTGTTGTTGTGTGAAGA |
| CD44 exon7 | Forward | TACGTCTTCAAATACCATCTCAGC |
|  | Reverse | ATCATCAATGCCTGATCCAGAA |
| CD44 exon8 | Forward | ACCACACCACGGGCTTT |
|  | Reverse | TCATCCTTGTGGTTGTCTGAAGTAG |
| CD44 exon9 | Forward | ATGTAGACAGAAATGGCACCAC |
|  | Reverse | TTGTGCTTGTAGAATGTGGGGTC |
| CD44 exon10 | Forward | TCCAGGCAACTCCTAGTAGTAC |
|  | Reverse | CAGCTGTCCCTGTTGTCGAATG |
| CD44 exon11 | Forward | TCAGCTCATACCAGCCATC |
|  | Reverse | CTTGATGACCTCGTCCCAT |
| CD44 exon12 | Forward | TATGGACTCCAGTCATAGTATAACGC |
|  | Reverse | CGTTGTCATTGAAAGAGGTCCTG |
| CD44 exon13 | Forward | AGCAGAGTAATTCTCAGAGCTTC |
|  | Reverse | TCAGAGTAGAAGTTGTTGGATGG |
| CD44 exon14 | Forward | GATGTCACAGGTGGAAGAAGAG |
|  | Reverse | ACTGCAGTAACTCCAAAGGAC |
| CD44 total | Forward | CTCTTGGCCTTGGCTTTGAT |
|  | Reverse | TTTCTGTCCTCCACAGCTCC |
| KHDRBS3 | Forward | GCTGATTACTATGATTACGGACATG |
|  | Reverse | GTCTTGAGTTAGTCCACTCTTC |

**Supporting table S3.** Sequences for analysis of CD44 isoform expression

| Designed at | Direction | Sequence (5’ 🡪 3’) |
| --- | --- | --- |
| Exon 5 | Forward | CTATTGTTAACCGTGATGGCACC |
| Exon 15 | Reverse | GGGGTGGAATGTGTCTTGGTCTC |
